# Neighborhood Inequalities in Retailers’ Compliance With the Family Smoking Prevention and Tobacco Control Act of 2009, January 2014–July 2014

**DOI:** 10.5888/pcd12.150231

**Published:** 2015-10-08

**Authors:** Joseph G. L. Lee, Hannah M. Baker, Leah M. Ranney, Adam O. Goldstein

**Affiliations:** Author Affiliations: Hannah M. Baker, Leah M. Ranney, Adam O. Goldstein, Tobacco Prevention and Evaluation Program, Department of Family Medicine, University of North Carolina School of Medicine, The University of North Carolina at Chapel Hill, Chapel Hill, North Carolina. Joseph G. L. Lee was employed at The University of North Carolina at Chapel Hill at the time this study was conducted.

## Abstract

**Introduction:**

Retailer noncompliance with limited US tobacco regulations on advertising and labeling was historically patterned by neighborhood in ways that promote health disparities. In 2010, the US Food and Drug Administration (FDA) began enforcing stronger tobacco retailer regulations under the Family Smoking Prevention and Tobacco Control Act of 2009. However, recent research has found no differences in compliance by neighborhood characteristics for FDA advertising and labeling inspections. We sought to investigate the neighborhood characteristics associated with retailer noncompliance with specific FDA advertising and labeling inspections (ie, violations of bans on self-service displays, selling single cigarettes, false or mislabeled products, vending machines, flavored cigarettes, and free samples).

**Methods:**

We coded FDA advertising and labeling warning letters (n = 718) for type of violations and geocoded advertising and labeling inspections from January 1 through July 31, 2014 (N = 33,543). Using multilevel models, we examined cross-sectional associations between types of violations and neighborhood characteristics previously associated with disparities (ie, percentage black, Latino, under the poverty line, and younger than 18 years).

**Results:**

Retailer advertising and labeling violations are patterned by who lives in the neighborhood; regulated tobacco products are more likely to be stored behind the counter as the percentage of black or Latino residents increases, and single cigarettes are more often available for purchase in neighborhoods as the percentage of black, poor, or young residents increases.

**Conclusion:**

Contrary to previous null findings, noncompliance with FDA advertising and labeling regulations is patterned by neighborhood characteristics, sometimes in opposite directions. Given the low likelihood of self-service violations in the same neighborhoods that have high likelihood of single cigarette sales, we suggest targeted approaches to FDA retailer inspections and education campaigns.

## Introduction

Regulation of tobacco products at the retail point-of-sale is an important component of de-normalizing use of tobacco products globally. The point of sale represents a major source of exposure to tobacco industry products and marketing in countries without strong regulation of this marketing channel, such as the United States. Retailers' marketing and compliance with regulations influence neighborhood health disparities through, for example, disproportionate exposure to tobacco marketing (1), low prices (2), promoting mentholated cigarettes (3), promoting little cigars (4), and having various levels of retailer compliance with regulations (5–15).

In the United States, stores that sell tobacco products (tobacco retailers) are regulated by the US Food and Drug Administration (FDA) under the Family Smoking Prevention and Tobacco Control Act of 2009 (FSPTCA). The FDA contracts with states to promote compliance and enforcement of 2 types of FSPTCA provisions, underage sales inspections and advertising and labeling inspections (16). Advertising and labeling regulations include bans on self-service displays (ie, customer-accessible products), sale of single cigarettes (or in packs of fewer than 20 cigarettes), vending machines in youth-accessible locations, certain product descriptors (eg, “light” and “mild”), flavored cigarettes (except menthol), and free samples of regulated tobacco products (www.fda.gov/TobaccoProducts/ResourcesforYou/Retail/default.htm). Under FDA regulations, the legal age for purchasing regulated tobacco products (ie, cigarettes, cigarette tobacco, and smokeless tobacco) in the United States is 18; however, one state (Hawaii) and approximately 80 localities have set higher ages (17).

Research is limited on neighborhood disparities in noncompliance with point-of-sale regulations other than underage purchases and single-cigarette sales. A 2014 systematic review identified only 7 studies worldwide reporting on the association of neighborhood characteristics and compliance with point-of-sale regulations on tobacco price, promotion, placement, and products (11). Of these, 3 examined sales of single cigarettes in the United States (8–10), 1 examined compliance with New Zealand's point-of-sale regulations (14), 1 examined Australian point-of-sale regulations (15), and 2 examined compliance with recent FDA regulations in a few geographic areas of the United States (12,13).

Before FDA regulation, noncompliance with the limited regulations for tobacco retailers was patterned in ways that promoted health disparities: merchants in communities with mostly Latino or black residents were more likely than merchants in white communities to sell cigarettes to any underaged buyer (7), particularly to underaged black and Latino buyers (6,7). Single cigarette sales by retailers were more common in neighborhoods with high percentages of low-income, black, or Latino residents than in neighborhoods with mostly white middle- or upper-income residents (8–10).

Recent research regarding compliance with the new FDA advertising and labeling regulations offers a conflicting view of neighborhood disparities. In Columbus, Ohio, Frick and colleagues found no differences between violations in high-income and low-income combined-zip-code areas after FDA regulation (12). In 3 counties in North Carolina, after implementation of FDA regulations, Rose and colleagues controlled for store type and neighborhood characteristics and found low likelihood of any violation in neighborhoods with a high percentage of black residents and high likelihood of violations in census tracts with a high percentage of families living under the poverty line (13).

By using publicly available FDA inspections data, we investigated whether specific advertising and labeling retailer violations (ie, false or mislabeled products or violations of bans on self-service displays, sale of single cigarettes, vending machines, flavored cigarettes, and free samples) were associated with particular neighborhood characteristics: the proportion of residents reporting black race, Latino ethnicity, income under the poverty line, or age under 18 years.

## Methods

We used the results from all FDA retailer advertising and labeling inspections (N = 33,543) from January 1 through July 31, 2014, that had been posted to the FDA's website by September 2, 2014 (www.accessdata.fda.gov/scripts/oce/inspections/oce_insp_searching.cfm, selected by choosing “minor involved = no”). The selected number of inspections is approximately 10% of all 324,859 FDA inspections and 21% of the 162,511 advertising and labeling inspections conducted from January 1 through July 31, 2014. One author (H.M.B.) coded warning letters (n = 718) for the violations described. Another author (J.G.L.L.) then independently coded 10% of warning letters and found no discrepancies. We were able to geocode all but 24 inspection locations and link them to census tract data. The Census Bureau designed census tracts to roughly approximate neighborhoods by using local committees to set boundaries (18), and we define neighborhoods as census tracts. We obtained census data for race (reported alone or in combination with other races), ethnicity, and age from the 2010 US Census (19); data for poverty status (for the population aged 18 years or older) were obtained from the 5-year estimates of the 2008–2012 American Community Survey (20). Poverty was defined as the percentage of people 18 or older with incomes under the US Federal Poverty Line in a given tract. For age, we used the percentage of people under age 18 in a given tract. Because small numbers can make demographic estimates unstable, we excluded 17 inspections in tracts with fewer than 100 residents. Poverty data were unavailable for 3 tracts.

Neighborhood characteristics were first calculated as the percentage of residents with a particular demographic characteristic (eg, 12% of census tract residents reporting black race, 15% of census tract population living under poverty line). For scaling purposes the percentage was then divided by 10 to create 10-percentage–point increments (eg, 12% = 1.2). Thus, a 1-unit change in these variables represents a 10-percentage–point increase in the neighborhood characteristic.

States are required to develop a formal sampling strategy that inspects 20% or more of the tobacco retailers in the state each year; however, states may implement their sampling plans and inspections in different ways (16). This variation in inspections presents 2 issues: 1) inspection results within states are more strongly correlated than results between states; and 2) the use of different (and not public) sampling strategies limits our ability to generalize outside this data source. To address the first issue of nonindependence of inspections within states, we used a multilevel modeling approach with random state intercepts and binary dependent variables (0 = no violation, 1 = violation). Each case was an inspection. Estimation was conducted with the adaptive quadrature method (9-point) by using PROC GLIMMIX in SAS 9.3 (SAS Institute, Inc). This second consideration limits the interpretation of our results to FDA inspections. Because of different sampling strategies, we did not report the prevalence of violations or compare violations between states, because results would be biased by any oversampling.

We created separate, unadjusted mixed models for predicting violations of self-service displays, single cigarette sales, false or mislabeled products, and vending machines for each neighborhood demographic characteristic. We found too few violations for flavored cigarettes and free samples to permit modeling of these outcomes. We then ran models adjusting for all neighborhood characteristics.

We set our significance level at *P *= .05 and used 2-tailed tests. Because we use only publicly available inspection results and census data, no human subjects were involved in this study.

## Results

At the time of analysis, 718 warning letters were posted on FDA's website for the period analyzed (January 2014–July 2014). Warning letters could be for more than 1 violation, but most (n = 689) letters analyzed were for only 1 violation; the remaining 29 were for 2 violations. The most common violation was for self-service displays of tobacco products (n = 553), followed by selling single cigarettes (n = 98), vending machines (n = 46), false or mislabeled products (usually relating to a vending machine) (n = 42), flavored cigarettes (n = 5), and free samples (n = 3).

Retailer advertising and labeling violations of the FSPTCA did not all occur in the same types of neighborhoods. Indeed, in unadjusted analyses, the likelihood of a self-service display violation was negatively associated with the proportion of black or Latino residents in neighborhoods (Table 1). Vending machines and mislabeled products, which are primarily cited as vending machines with “light” or “mild” labels, were negatively associated with the percentage of buyers under age 18. In contrast, the likelihood of a single-cigarette violation was positively associated with the proportion of black and under-18 residents as well as the proportion of adult residents with incomes under the poverty line.

**Table 1:** Unadjusted Relationship Between FDA Tobacco Retailer Advertising and Labeling Violations and Neighborhood Characteristics, January 2014–July 2014

Model^a^		Self-Service Display	Sale of Single Cigarettes	False or Mislabeled Cigarettes	Vending Machine Sales
% Black (10s)	Intercept^b^ (SE)	0.01 (0.31)	<0.01 (0.80)	<0.01 (0.35)	<0.01 (0.32)
	OR (95% CI)	0.81 (0.76-0.87)	1.37 (1.26-1.48)	0.92 (0.75-1.11)	0.88 (0.71-1.08)
% Latino (10s)	Intercept^b^ (SE)	<0.01 (0.32)	<0.01 (0.79)	< 0.01 (0.35)	< 0.01 (0.32)
	OR (95% CI)	0.92 (0.85-0.99)	1.13 (0.96-1.33)	0.79 (0.58-1.09)	0.95 (0.78-1.15)
% Poverty (10s)	Intercept^b^ (SE)	0.01 (0.32)	< 0.01 (0.78)	<0.01 (0.42)	<0.01 (0.39)
	OR (95% CI)	0.97 (0.79-0.96)	1.54 (1.31-1.81)	1.11 (0.83-1.47)	1.16 (0.90-1.51)
% Age 18 or younger (10s)	Intercept^b^ (SE)	0.01 (0.37)	<0.01 (0.95)	<0.01 (0.56)	<0.01 (0.55)
	OR (95% CI)	0.92 (0.78-1.09)	1.70 (1.11-2.59)	0.41 (0.26-0.64)	0.44 (0.28-0.69)
ICC^c^		0.53	0.68	0.33	0.34

Abbreviations: CI, confidence interval; ICC, intra-class correlation; OR, odds ratio; SE, standard error.

a N = 33,499 for percentage of the poverty model; n= 33,502 for other models because of missing census data. "10s" indicates variables scaled to represent 10-percentage point increments (eg, 12% = 1.2).

b Intercept is reported as exponentiated (ie, odds for the average of demographic characteristics).

c ICC was calculated as ICC = τ_00_ / (τ_00_ + [π^2^ / 3]) and should be interpreted as the ICC for a hypothetical latent continuous variable underlying the binary variable.

To determine whether the neighborhood characteristics we examined (ie, percentage who were black, were Latino, were younger than 18, or had incomes under the Federal Poverty Line) independently predicted FSPTCA violations or whether they confounded each other, we modeled adjusting for these percentages. When we simultaneously controlled for percentage black, Latino, incomes under the Federal Poverty Line, and age under 18 years, the percentage black and Latino held significance for self-service displays, and poverty and younger than 18 were no longer significant for single cigarette sales (Table 2). When race and ethnicity are statistically held constant, neighborhood poverty is not a significant predictor of violations.

**Table 2:** Adjusted Relationship Between FDA Tobacco Retailer Violations and 2010 Neighborhood Characteristics, January 2014–July 2014, n = 33,499

Characterisitic	Self-Service Display	Sale of Single Cigarettes	False or Mislabeled Cigarettes	Vending Machine Sales
Intercept^a^ (SE)	0.01 (0.39)	<0.01 (0.91)	<0.01 (0.72)	<0.01 (0.69)
% Black, OR (95% CI) (10s)	0.80 (0.74-0.86)	1.38 (1.23-1.54)	0.93 (0.75-1.15)	0.88 (0.70-1.11)
% Latino, OR (95% CI) (10s)	0.90 (0.83-0.99)	1.24 (1.02-1.52)	0.88 (0.63-1.23)	1.03 (0.84-1.28)
% Poverty, OR (95% CI) (10s)	1.06 (0.95-1.19)	1.13 (0.91-1.39)	1.04 (0.77-1.40)	1.10 (0.83-1.44)
% Under 18, OR (95% CI) (10s)	1.11 (0.92-1.35)	0.86 (0.54-1.36)	0.44 (0.26-0.75)	0.46 (0.27-0.78)

Abbreviations: CI, confidence interval; OR, odds ratio; SE, standard error.

a Intercept is reported as exponentiated (ie, odds for the average of demographic characteristic). "10s" indicates variables scaled to represent 10-percentage point increments (eg, 12% = 1.2).

## Discussion

Noncompliance with the FSPTCA is patterned by neighborhood characteristics, sometimes in opposite directions for different types of violations. Our analysis provides evidence that sales of single cigarettes are more likely to occur in neighborhoods as the proportions of black, poor, or young residents increase. Self-service displays are less likely to be found in neighborhoods as the proportions of Latino or black residents increase. When adjusted for neighborhood characteristics, as the proportion of black and Latino residents increases, the likelihood of a self-service display violation decreases while the likelihood of a single-cigarette sale violation increases, indicating that race and ethnicity are not simply proxies for neighborhood poverty in this relationship. Vending machines are less likely in neighborhoods as the percentage of youths under age 18 increases, again after controlling for neighborhood characteristics.

Our findings differ from those of 2 previous studies of neighborhood disparities in retailer compliance with advertising and labeling regulations that found no evidence of disparities (12,13). We believe these differences stem from 2 methodological approaches (12,13). First, previous research audits of tobacco retailers used data collectors who did not have FDA authority and possibly had less access than FDA inspectors to areas where violations were more likely to be found. For example, after an intervention to address retailer sales of single cigarettes, Woodruff and colleagues found that many retailers simply moved singles to a hidden location behind the counter (21). Second, research audits of FDA compliance analyzed violation types in aggregate; if certain violations were found in different types of neighborhoods, as our findings suggest, this would attenuate any overall effect.

Self-service displays of tobacco products are associated with greater access to tobacco products by customers under age 18 (22). Retailers may remove self-service displays because of real or perceived shoplifting (23). Thus, it is possible that racially biased perceptions of crime in neighborhoods with high percentages of Latino or black residents could reduce the presence of self-service displays. Indeed, in a large study of urban inequality, many retailers indicated they conflated neighborhood racial composition with crime and incorporated these perceptions into management decisions (24).

Single-cigarette sales facilitate tobacco initiation by young people, may allow retailers to avoid taxes, and do not have warning labels. Historically single cigarettes are available from retailers even in areas with a ban on their sale (9), and our findings mirror earlier research showing high availability of single cigarette sales in neighborhoods with a high percentage of black residents (9). Reasons for this disparity are unclear. There may be market demand for singles: in a 2005–2008 survey of participants in an employment and job training program in Baltimore, Maryland, 60% of respondents reported retailers were likely to sell singles, and half of respondents thought single cigarettes should be available (25). Educational campaigns to reduce single-cigarette sales by retailers show some evidence of success but may also cause retailers to sell singles more clandestinely (21).

Historically vending machines are used more frequently by younger teenagers than by older teenagers (26). However, survey research indicated few teenagers typically bought cigarettes from vending machines; this research also showed more white male teenagers bought cigarettes from vending machines than did black or female teenagers (27). Our experience suggests that identifying vending machine violations is largely a vestige of the pre-FSPTCA era and is primarily relevant for small, independent stores. Regardless, our finding with regard to vending machine violations is encouraging: they are more likely in neighborhoods as the percentage of people younger than 18 decreases.

There are important limitations to this research. First, states can use different sampling strategies, but the FDA does not release sampling weights, which limits our ability to interpret the prevalence of violations and to generalize beyond FDA inspections. Second, our data cover all advertising and labeling inspections for 7 months and may not represent other periods. Third, because this is a cross-sectional study, we cannot infer causality between neighborhood demographics and retailer violations. Fourth, we do not have access to information regarding store type and therefore cannot assess the role of store type in these findings. Low-income neighborhoods with diverse residents have more small stores and non-chain stores than homogeneous neighborhoods (28). Nonetheless, we used publically available data from inspections during which inspectors have better access than researchers to store data, and our modeling strategy accounts for within-state similarity between inspections.

Future work should explore the origins of retailer violations, including retailer knowledge of regulations, the role of store type, and management decisions based on neighborhood characteristics. Overall odds of an advertising and labeling violation were low, 0.01 or lower in our models, and researchers that adapted inspection protocols to reflect real-world attempts by young people to purchase cigarettes (29) may need to modify those protocols in order to produce data that could be used to improve procedures for advertising and labeling inspections.

Tobacco retailer violations of the FSPTCA are patterned by neighborhoods in such a way as to promote the production and continuation of disparities in tobacco use, thereby perpetuating disproportionate preventable tobacco-related disease, disability, and death. Youths in neighborhoods with high percentages of black, poor, or young residents may have single cigarettes more available to them than youths in neighborhoods with mostly white, affluent, or older residents. Conversely, youths in neighborhoods with mostly white residents may be more exposed to self-service displays than are youths in neighborhoods with high percentages of black and Latino residents. FDA enforcement of the FSPTCA should address these neighborhood disparities when developing inspection sampling plans, retailer education programs, and targeted enforcement.

We note that research on youths’ access to tobacco shows clearly that policy without enforcement is ineffective (30). FDA's enforcement of FSPTCA regulations through advertising and labeling inspections is an important part of reducing tobacco use and may help reduce disparities in tobacco retailer compliance through careful retailer education and targeted enforcement.
